# Effects of Marketing Ages on the Physicochemical Properties and Sensory Aspects of Cured Broiler Chicken Breast Meat

**DOI:** 10.3390/foods10092152

**Published:** 2021-09-12

**Authors:** Sin-Young Park, Hack-Youn Kim

**Affiliations:** Department of Animal Resources Science, Kongju National University, Yesan-Gun 32439, Chungnam, Korea; sinsu1225@gmail.com

**Keywords:** broiler chicken, chicken breast, cured meat products, marketing age, quality properties

## Abstract

This research evaluated the properties of cured chicken breasts of broiler chicken with different marketing ages (28, 30, 32, and 34 day). The water contents in the proximate compositions of the samples tended to decrease with increasing marketing age, while the protein content increased. The samples’ uncooked and cooked pH values, WHC, and cooking yield increased with increasing marketing age; however, the WHC and cooking yield were not significantly different between the 32 and 34 day samples (*p* > 0.05). In the case of the color, the 34 day samples were significantly lower in terms of lightness, but significantly higher in redness and yellowness compared to the other samples (*p* < 0.05). Although the shear forces of the 28–32 day samples were not significantly different (*p* > 0.05), those of the 28 and 30 day samples were significantly lower than those of the 34 day sample (*p* < 0.05). Furthermore, the aromatic profile (determined by principal component analysis) of the 34 day sample differed from that of the 28–32 day samples. Flavor evaluation of the cooked 30 and 32 day samples showed significant differences compared to the 28 and 34 day samples (*p* < 0.05), and the texture evaluation showed that the 34 day sample obtained a significantly lower score than the 28 day sample (*p* < 0.05). Overall, these results suggest that the current broiler marketing age of 32 day results in suitable quality properties for broiler cured chicken breast.

## 1. Introduction

The poultry industry worldwide has grown significantly, and the production index of broiler chickens (*Gallus gallus domesticus*) has also improved, leading to the greatly improved daily weight gain of broiler chickens [1]. In regards to the broiler production indicators in South Korea, the average carcass weight of chickens with a marketing age of 35 day was 1.6 kg in 2011, whereas the average carcass weight of chickens with a marketing age of 34 day was 1.9 kg in 2020, which represents a clear improvement [2,3]. Therefore, at the same marketing age, the quality characteristics have recently been significantly improved compared to the past. Furthermore, upon comparison with the 2003 growth curve of worldwide broiler chickens, it was apparent that the carcass size was significantly smaller than that of the current general marketing age (30–36 day) due to a relatively lower productivity [4], and the breasts, legs, and wings were not large enough to be used as retail cuts. However, with improvements in the production index of broiler chickens, the sizes of these retail cuts have continuously improved. As an example, in the 1940s, 104 g of breast meat was obtained from a chicken carcass with a marketing age of 17 weeks (84 day) [5], while in the 1990s, 190–233 g of breast meat was obtained from a chicken carcass with a marketing age of 7 weeks (49 day) [6]. This has been improved further in recent years, with 196–230 g of breast meat being obtained from a chicken carcass with a marketing age of 28–30 day in 2020, thereby confirming the significant improvement in terms of the daily weight gain of retail cuts [3].

However, despite such improvements, the problem of low economic efficiency due to high production costs still remains, since these retail cuts (breast, legs, and wings) are separated from broiler chickens with a higher marketing age [7]. As a result, the texture and flavor properties of these cuts obtained from higher marketing age chickens (36 day or higher) are less favorable compared to those of broiler chickens with a common marketing age (28–32 day) [8]. Due to overall higher expectations in terms of food quality, consumers are demanding greater quality meat and processed meat products [9], and therefore it is necessary to assess the processing suitability of retail cuts (breasts, legs, and wings) obtained from broiler chickens with a common marketing age, since these cuts will be expected to present superior organoleptic properties compared to broiler chickens with a 36 d or higher marketing age.

With respect to the retail cuts of broiler chickens, the demand for chicken breast has increased in recent years since it is commonly consumed during weight loss or exercise programs. Chicken breast is a high protein meat with a very low fat content and a high protein content compared to other meats and retail cuts. This high protein content provides a feeling of satiety and aids in muscle building [10,11]. However, it also contributes to a poor flavor and undesirable texture if no additives (e.g., salt or spices) are added to improve the organoleptic properties, or if the meat is not processed [12]. Since various processed chicken breast products are currently available on the market, the use of additives to maximize the characteristics of chicken breasts should be minimized in terms of the processed chicken breast products to maximize their health benefits [13]. To maximize the effects of small amounts of additives in the industrial processing of chicken breast, cured meat products are produced by heating after curing through a tumbling process to effectively penetrate the chicken breast with the prepared curing solution [14]. The organoleptic properties of cured chicken breast prepared in this way are therefore enhanced, even when minimal levels of additives are employed, thereby enabling the consumption of cured chicken breast as a healthy food.

The aim of this study is therefore to determine the marketing age that results in optimal quality characteristics. This is achieved by manufacturing cured chicken breast samples with various marketing ages and analyzing the resulting physicochemical and organoleptic properties.

## 2. Materials and Methods

### 2.1. Cured Chicken Breast Manufacture

For the manufacture of cured chicken breast with various marketing days (28, 30, 32, and 34 day), Ross broiler (*Gallus gallus domesticus*) skinless breast muscle (pectoralis major muscle) was provided by a local farm (Chungnam, Korea) after slaughter. The skinless chicken breast for each marketing day sample was removed from the visible connective tissue and fat tissue of the meat surface. The curing solution employed for the cured chicken breast was composed of 83.5% ice water, 1% tripolyphosphate, 7.5% nitrite pickling salt (99.4% NaCl and 0.6% nitrite), 3.5% white sugar, and 4.5% curing agent (00770 Jambolak Gold, Sewoo Inc., Gyeonggi, Korea). These components were mixed thoroughly to ensure complete dissolution, and the prepared chicken breast was placed in a tumbler (BVBJ-40, Thematec, Gyeonggi, Korea) with the obtained curing solution in a ratio of 1:4, then tumbled at 22 rpm for 1 h. After the tumbling process, the chicken breast and remaining curing solution were placed in a refrigerator (CA-H17DZ, LG, Seoul, Korea) at 4 °C for 24 h. Subsequently, the cured chicken breast samples were thermally processed in a cooking chamber (10.10 ESI/SK, Alto Shaam, Menomonee Falls, WI, USA) at 80 °C for 40 min and then cooled at room temperature (20 °C) for 30 min.

### 2.2. Proximate Composition

The proximate composition (moisture, protein, fat, and ash contents) was measured by following the method of the Association of Official Analytical Chemists (AOAC) [15]. The moisture content was measured using the oven-drying method, the crude protein content was measured according to the Kjeldahl method, the crude fat content was measured using the Soxhlet method, and the ash content was measured according to the dry-ashing method.

### 2.3. pH

The pH of each sample was measured using a pH meter (Model S220, Mettler-Toledo, Schwerzenbach, Switzerland). More specifically, each sample (4 g) was homogenized in distilled water (16 mL) using a homogenizer (HMZ-20DN, Poolim Tech, Seoul, Korea) at 8000 rpm for 1 min, after which, the sample’s pH was measured.

### 2.4. Water-Holding Capacity (WHC)

The water holding capacities (WHCs) of the uncooked cured chicken breast samples were determined using the slightly modified filter paper press method [16] with slight modifications. More specifically, each uncooked cured chicken breast sample (300 mg) was placed on a filter paper and was compressed for 3 min using a plexiglass plate device. The WHC was then calculated from the meat and exudation areas as follows:

$$\mathrm{WHC}\;\left(\%\right)=\frac{Meat\;area\;\left(mm^{2}\right)}{Exudation\;area\;\left(mm^{2}\right)}\times 100.$$

### 2.5. Curing Yield

The curing yield of the cured chicken breast was determined by calculating the difference in the cured chicken breast weight before and after curing, as follows:

$$\mathrm{Curing}\;\mathrm{yield}\;\left(\%\right)=\frac{Weight\;after\;curing\;\left(g\right)}{Weight\;before\;curing\;\left(g\right)}\times 100.$$

### 2.6. Cooking Yield

The uncooked cured chicken breast was weighed and cooked in an 80 °C chamber for 40 min. After subsequent cooling at 10 °C for 1 h, the cooked samples were weighed, and the cooking yield was calculated using the following formula:

$$\mathrm{Cooking}\;\mathrm{yield}\;\left(\%\right)=\frac{Weight\;after\;cooking\;\left(g\right)}{Weight\;before\;curing\;\left(g\right)}\times 100$$

### 2.7. Color

The CIE color of the cured chicken breast surface was measured using a color meter (CR-10 plus, Minolta, Tokyo, Japan; A white standard plate used as a reference: CIE L*, +96.3; CIE a*, 0.0; CIE b*, +2.0) to measure the lightness (L*), redness (a*), and yellowness (b*) values. Hue angle (H°) of samples were calculated using the following equation: tan^−1^(b*/a*), and the Chroma value of samples (C*) were calculated using the following equation: (a*^2^ + b*^2^)^1/2^.

### 2.8. Shear-Force

The shear-force of each cooked cured chicken breast sample (2.0 cm × 1 ø; length × diameter) was measured using a TA 1 texture analyzer with attached V-blade (Ametek Inc., Berwyn, PA, USA) at a test speed of 2.0 mm/s, a distance of 2.2 cm, and a force of 5.6 N. The measured values are expressed in N.

### 2.9. Electronic Nose

The aroma profile of each cooked cured chicken breast sample was analyzed using a Heracles II electronic nose (Alpha MOS, Toulouse, France). The electronic nose headspace conditions were as follows: sample vial, 20 mL; sample volume, 5 g; heating temperature, 60 °C; carrier gas, humidified synthetic air; flow rate, 250 mL/min; injection volume, 2.5 mL; acquisition time, 120 s. During the retention time (230 s) of each sample, the intensity peak of the aroma substance was found. After that, the distinguished principal component (aroma substance) of the samples was taken as the primary component (PC1) and secondary component (PC2) values. For the classified aroma profiles, Alpha Soft software (Alpha MOS, Toulouse, France) was used.

### 2.10. Electronic Tongue

The taste profiles of each cooked cured chicken breast sample were measured using an Astree electronic tongue (Alpha MOS, Toulouse, France). To measure the sample sourness, saltness, and umami, 0.1 M HCl, 0.1 M NaCl, and 0.1 M MSG were used as reference materials for the electronic tongue sensor, respectively. The ground samples were (4 g) mixed with 16 mL of distilled water and homogenized 10,000 rpm for 1 min using an Ultra-Turrax homogenizer. The homogenate was filtered. The taste profile of the filtrate (diluted with distilled water in the ratio of 1:100) was analyzed using an electronic tongue (acquisition duration, 120 s; acquisition period, 1 s). The taste profiles were analyzed using the Alpha software program (Alpha MOS, Toulouse, France) and the 7-sensor array (Ref 803-0175; Astree electronic tongue) was expressed as AHS (sourness), PKS, CTS (saltiness), NMS (umami), CPS, ANS, and SCS.

### 2.11. Sensory Evaluation

The sensory evaluation of each sample was performed by the sensory evaluation method of Kim and Kim [17]. Twenty-five sensory panelists (male: 10, female: 15, age: 21–32) used a basic taste identification test and were trained with commercial cured chicken breast products to ensure familiarization with the sensory properties of the cured chicken breast to be evaluated. The color, flavor, texture, juiciness, and overall acceptability of the samples were evaluated based on a 10-point descriptive scale (color: 1 = Extremely desirable, 10 = Extremely undesirable; flavor: 1 = extremely inadequate, 10 = extremely adequate; texture: 1 = extremely tough, 10 = extremely tender; juiciness: 1 = extremely dry, 10 = extremely juicy; overall acceptability: 1 = Extremely unacceptable, 10 = Extremely acceptable). Sensory evaluation was approved by the Kongju National University’s Ethics Committee (Authority No: KNU_IRB_2020-40).

### 2.12. Statistical Analysis

All analyses, with the exception of the aromatic profiling (PCA) results, were assessed after carrying out each measurement in triplicate (minimum). A statistical analysis of variance was performed on all variables compiled and analyzed by the General Linear Model using SAS version 9.3 (SAS Institute, Cary, NC, USA), and Duncan’s multiple range test was performed to verify the significance of differences (*p* < 0.05). The data are presented as means ± standard error (SE).

## 3. Results and Discussion

### 3.1. Proximate Composition

Table 1 outlines the results of proximate composition measurements for the cooked cured chicken breast samples with various marketing ages. As indicated, the crude fat and crude ash contents did not show any significant differences between different marketing ages (*p* > 0.05), although the water content tended to decrease with increasing marketing age, and the crude protein content increased. In general, livestock used for meat tends to develop more muscle with increasing age, which results in the development of intramuscular connective tissues, such as collagen, elastin, and reticulin, and leads to a meat product with a denser structure [18]. Reticulin is a histological term used to describe a type of fiber in connective tissue composed of type 3 collagen in which these reticular fibers crosslink to form a fine meshwork [18]. Collagens are a large family of proteins that are widely distributed in nature with a simple, repetitive sequence of amino acids that serves as a defining signature for the proteins [19]. Elastin is a protein that exists as fibers in the extracellular spaces of many connective tissues. Elastin derives its name from its ability to act like an elastic band, that is, to stretch and recoil with transient force [18]. This accounts for the increased protein content and the reduced water content observed herein with increasing marketing age. According to a recent study of the characteristics of broiler carcasses, among the various changes in the proximate composition, the protein content tended to increase with increasing marketing age, with a higher protein content being found in the 34 day sample than in the 28 day sample, as also observed herein [3]. Such differences in protein content according to age were also consistent with the results of carcass characteristics analysis in the 30–50 day samples examined by Abougabla and Taboosha [20], who also reported that the protein content increased while the water content decreased. These results indicate that the differences in the proximate composition according to the marketing age would also affect the proximate composition of cured chicken breast. However, in contrast, Berri et al. [21] reported that the protein content decreased in broiler chickens over 6 weeks of age, despite some differences being found depending on the feeding method and dietary nutrients consumed. This was thought to be caused by fat accumulation upon completion of the initial maturation stage. Although the daily weight gain of broiler chickens varies with age, the maximum daily weight gain tends to be achieved between days 30 and 40 and, following muscle formation, the daily weight gain gradually decreased with increasing fat accumulation [22]. Therefore, the 30–34 day sample appears to be optimal in terms of its potential use as a high protein source. It should also be noted that in recent years, with the gradual decrease in the marketing age, chickens are slaughtered at a point where their muscle connective tissues are still in an immature state, which results in a lack of cohesiveness between the meat tissues, and an insufficient firmness during the processing of retail cuts [23]. It is therefore necessary to set an appropriate marketing age suitable for processing.

### 3.2. pH and Water Holding Capacity (WHC)

Table 2 lists the pH and WHC properties of the uncooked and cooked cured chicken breast samples with various marketing ages. As shown, the pH of uncooked cured chicken breast did not present any significant differences between marketing ages of 28–30 day (*p* > 0.05), but a significant increase was observed after 32 day (*p* < 0.05). Similarly, the pH of the cooked cured chicken breast also significantly increased with an increase in the marketing age (*p* < 0.05). As expected based on these results, the WHC of the uncooked cured chicken breast with a marketing age of 30–34 day was significantly higher than that of the uncooked cured chicken breast with a marketing age of 28 day (*p* < 0.05). Połtowicz and Doktor [24] also reported that the pH of broiler chicken breast with a higher marketing age (70 and 84 day) was higher than that of chicken breast with a relatively lower marketing age (56 day).

In chicken breast, the muscle weight and glycogen content are negatively correlated, and muscles with a low glycogen content generally have a high pH when converted to meat [25]. This accounts for our observations that cured chicken breast with a higher marketing age exhibited a higher pH, wherein the WHC also increased with increasing pH. Since chicken meat with a high WHC exhibits reduced water loss during cooking and becomes juicier during processing [26], the appropriate marketing age for cured chicken breast processing was considered to be 30–34 day. Muscle fibers are traditionally classified as type I (slow-twitch oxidative), IIA (fast-twitch oxidative glycolytic), and IIB (fast-twitch glycolytic) [27,28,29]. Broiler chicken mostly consists of Type II white meat with a uniform and dense meat structure [23]. Due to this denser structure, it is necessary to perform curing and tumbling processes during the manufacture of cured meat products based on chicken breast, to ensure that the curing solution can penetrate deep into the chicken breast. The curing solution should not be exuded during heating, cooling, or storage after the tumbling process [14]. In this context, the water holding capacity (WHC) of a meat sample refers to its ability to control the exudation of the curing solution or chicken breast juice, and so raw meat with an excellent WHC should be used when manufacturing cured chicken breast [30]. It should also be noted that pH is an important factor in determining the WHC. The composition of positive and negative charges on the meat components varies depending on the pH. More specifically, upon increasing the number of similar electric charges, greater numbers of repulsive forces result in more space within the protein structure, thereby increasing the WHC [30]. The high WHC of uncooked cured chicken breast prevents exudation of the curing solution or juice that may form during cooking, and the high WHC of cooked cured chicken breast prevents loss during storage [31].

### 3.3. Curing and Cooking Yield

**Figure 1:**
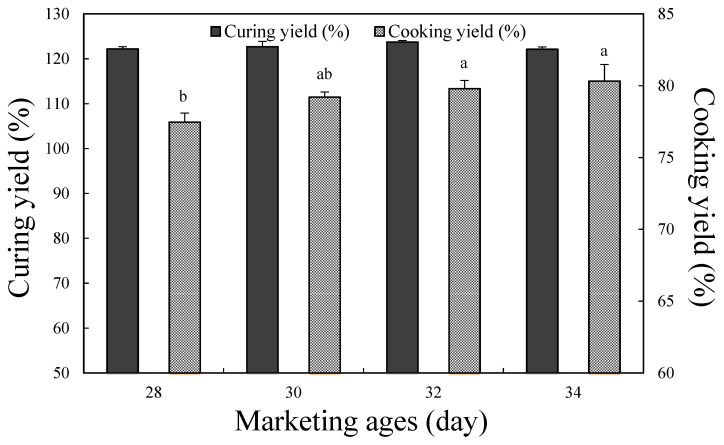
Curing yield and cooking yield of cured chicken breast with various marketing ages. ^a–b^ Means on the same bar with different letters are significantly different (*p* < 0.05).

The curing yield in non-crushed cured meat products refers to the ratio at which the curing solution penetrates into the muscle fibers, and it has a close correlation with the economy and quality of cured meat products. Products with a high curing yield can hold a large amount of curing solution, which leads to a high yield of the final product. In addition, the additives present in the curing solution are also spread evenly inside the meat, thereby enhancing the usability of the additives [32]. For the purpose of this study, the curing solution was not injected during preparation of the cured chicken breast, but the chicken breast was cured for 24 h after vacuum-tumbling in the curing solution. Figure 1 shows the curing yield after the curing process, wherein no significant differences were observed according to the marketing age (*p* > 0.05). Remarkably, even without the use of an injection process to facilitate diffusion of the curing solution, the curing yield ranged from 122.13% to 123.72%, thereby indicating the importance of the WHC of cured chicken breast in retaining the curing solution without exudation.

The cooking yield of the cured chicken breast is also presented in Figure 1. More specifically, the cooking yield tended to increase with age up to a marketing age of 32 day, with a significantly higher yield being achieved at 32 day than at 28 day (*p* < 0.05); however, no significant differences were observed between the marketing ages of 30 and 34 day (*p* > 0.05). Therefore, in terms of the cooking yield, chicken breast with a marketing age of 30–34 day is suitable for the manufacture of cured chicken breast. In broiler chickens, the cross-sectional area of the muscle fibers increases with age, which in turn increases the resistance to the external physical environment, thereby resulting in drip losses and cooking losses [25]. These results further confirm the previously reported relationship between the cooking yield and the WHC [31,33,34]. Therefore, cured chicken breast with a marketing age of 30–34 day, which exhibits a large cross-sectional muscle area and a high WHC, gave an excellent cooking yield.

### 3.4. Color

Table 3 lists the color properties of cooked cured chicken breast of various marketing ages. As indicated, there was no significant difference in the lightness between the marketing ages of 28 and 30 day, but a significant decrease was observed after 32 day (*p* < 0.05). In addition, the redness of cured chicken breast with a marketing age of 34 day was significantly higher than that of the other samples (*p* < 0.05), and the yellowness increased with an increase in the marketing age. In general, the color of meat changes according to its age due to the fact that the myoglobin content, that is, the pigment protein present in meat, increases with age [35]. Even if white muscle fiber is dominant in the chicken breast, changes in the meat color can occur as the ratio of red muscle fiber increases slightly with age [3]. In this study, nitrite pickling salt (99.4% NaCl, 0.6% nitrite) was added during preparation of the curing solution to produce nitrosomyoglobin, which is converted to nitrosohemochrome upon heating, and is responsible for the color of the cured meat [36]. To set an optimal color for processed cured chicken breast, hue angle and chroma value were calculated, derived from redness and yellowness. The hue angle of 34 day was significantly lower than that of the other marketing ages (*p* < 0.05), and the chroma value of 34 day was significantly higher than that of the other marketing ages (*p* < 0.05). These results suggest that a marketing age of 34 day seems to have a different color than other marketing ages. Thus, marketing ages of 34 day represent different chromaticity, and therefore judgements about differences in apparent quality may occur.

### 3.5. Shear-Force

**Figure 2:**
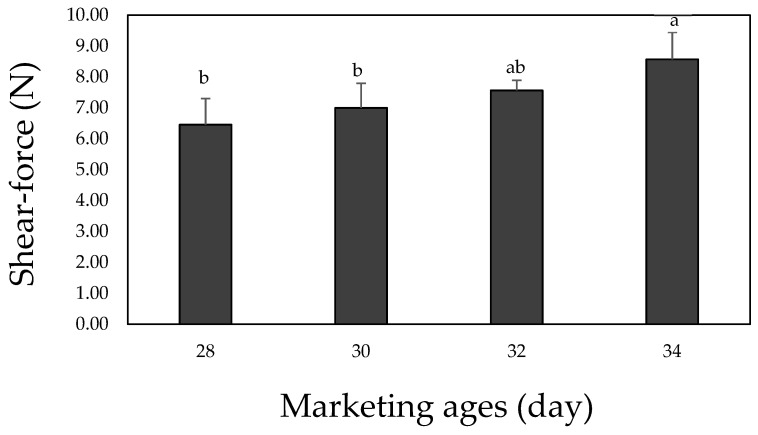
Shear-force of cured chicken breast with various marketing ages. ^a–b^ Means on the same bar with different letters are significantly different (*p* < 0.05).

Figure 2 shows the shear force measurement results for the cured chicken breast with various marketing ages, wherein it is apparent that the shear force tended to increase with increasing marketing age after the age of 30 day. Although the shear force was significantly higher at the age of 34 day than at 28 and 30 day (*p* < 0.05), no significant difference was observed between 28–32 day (*p* > 0.05). In general, the shear force of meat increases with age due to the fact that the protein content in the muscle increases relative to the other components, and the composition of the connective tissue becomes denser as the livestock matures [36]. Such densification of the muscle protein is caused by changes in the properties and collagen and elastin content, that is, the proteins of which the connective tissue is composed [20]. In the context of collagen, which has the most significant effect on reducing the protein’s tenderness upon maturation, the degree of crosslinking between the polypeptide chains increases, resulting in the formation of a more robust structure [18]. Therefore, even considering the same breed, variety, sex, and feeding environment, older organisms tend to possess a more robust meat protein structure. This explains the observed increase in the shear force after 30 day. In this context, Wasserman [37] examined the age-specific properties of broiler chicken meat stored at the same temperature, and reported that the shear force at the age of 6 weeks was higher than that at the age of 5 weeks. Park et al. [3] also reported that the shear forces of broiler chicken breast and leg samples were higher in older organisms.

### 3.6. Aromatic and Taste Profiles

**Figure 3:**
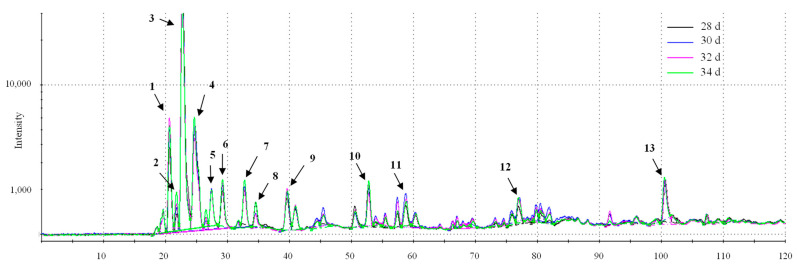
Volatile compounds of cooked cured chicken breast with various marketing ages; 28 d: cured chicken breast with 28 days of marketing age; 30 d: cured chicken breast with 30 days of marketing age; 32 d: cured chicken breast with 32 days of marketing age; 34 d: cured chicken breast with 34 days of marketing age. Peaks are reported in order of elution: 1, acetaldehyde; 2, trimethylamine; 3, methyl formate; 4, 2-propanol; 5, 2-methylpropanal; 6, 2-propanol; 7, ethyl acetate; 8, butan-2-one; 9, 3-methylbutanal; 10, pyrrole; 11, hexanal; 12, octanal; 13, methyl eugenol.

**Figure 4:**
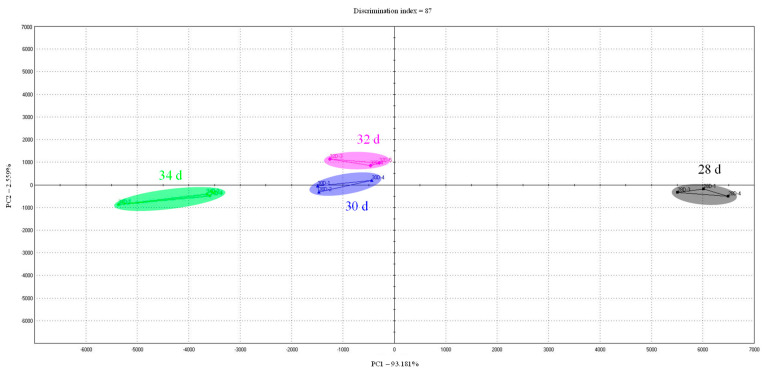
Principal component analysis of the aromatic profile of cooked cured chicken breast with various marketing ages; 28 d: cured chicken breast with 28 days of marketing age; 30 d: cured chicken breast with 30 days of marketing age; 32 d: cured chicken breast with 32 days of marketing age; 34 d: cured chicken breast with 34 days of marketing age.

To examine the differences in the volatile aroma of cooked cured chicken breast samples, the results of the analysis of volatile compounds according to the retention time for each sample were shown in chromatograms (Figure 3). In terms of volatile aroma compounds according to the marketing ages, 3-methylbutanal (Figure 3—peak 9) and hexanal (Figure 3—peak 11) were higher in 30, 32, and 34 day than in 28 day. The aroma characteristics of 3-methylbutanal are an almond, malty, and toasted, and hexanal is a fatty and tallowy aroma. Therefore, it is judged that cured chicken breast with a marketing age of 30, 32, and 34 day can exhibit the savory and oily aroma slightly higher than it does at 28 d. The case of 34 day showed higher levels of ethyl acetate (Figure 3—peak 8) and octanal (Figure 3—peak 12) than the other samples. As such, although the crude fat content did not show a significant difference according to marketing ages, the aroma related to the fat contents increased with increasing marketing ages. Therefore, 34 day is expected to have a slightly different aroma than 28, 30, and 32 day. In particular, octanal in meat and meat products, as the intensity increases, exhibits a rancid flavor, which can cause an off-flavor [38]. The results of the electronic nose analysis of chicken breast meat of various marketing ages expressed aromatic profiles as a PCA plot (Figure 4). In the PCA plot based on the electronic nose analysis, PC1 and PC2 are the indices for the degree of difference in terms of the aroma according to the position of the x-axis and the y-axis, respectively [17]. It was found that PC1 was 75.847% and PC2 was 10.261%, thereby indicating a large difference in aroma along the x-axis. Examination of this difference in terms of the marketing age showed that differences in aroma were recorded between 28 and 30 day, although no significant difference was observed between the ages of 30 and 32 day. However, at an age of 34 day, a significant difference in aroma was found for the cured chicken breast compared to that recorded between 28 and 32 day. In general, the compositions of free amino acids and nucleic acid-related substances that affect the meat aroma in livestock change with age [39,40]. Unlike other livestock, such as pigs and cows, chicken has a high ratio of white meat. In particular, breast meat is a representative source of white meat, which is low in fat [41]. It is also characterized by its high content of unsaturated fatty acids, although the composition of these unsaturated fatty acids in broiler chickens changes as they grow [40] due to their facile oxidation, which can result in an “off” flavor [42], and could account for the aroma changes detected herein with age. In particular, cured chicken breast at an age of 34 day possessed a different aroma from the 28–32 day samples, indicating the presence of a significant “off” flavor compared to the younger samples.

**Figure 5:**
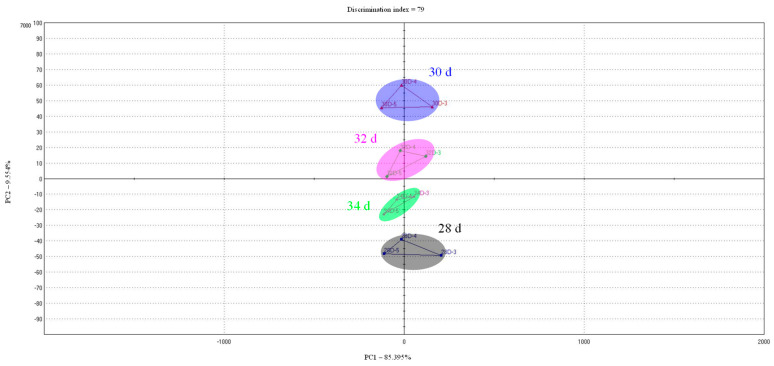
Principal component analysis for the taste profile of cooked cured chicken breast with various marketing ages; 28 d: cured chicken breast with 28 days of marketing age; 30 d: cured chicken breast with 30 days of marketing age; 32 d: cured chicken breast with 32 days of marketing age; 34 d: cured chicken breast with 34 days of marketing age.

Table 4 shows the taste score of the samples based on the electronic tongue analysis. Sourness did not show a constant change according to marketing ages, but saltiness and umami showed a tendency to increase as the marketing age increased. Similar to the experimental results of this study, there was a report that umami components, such as IMP, increased with age in chicken meat [43]. However, Jayasena et al. [44] confirmed that the IMP content in raw meat increased as the age of chickens increased, but it did not significantly affect the taste-active compounds of chicken meat. Accordingly, as a result of PCA analysis of the taste profile based on the taste score (Figure 5), PC1 showed 85.395% and PC2 9.554%, and it was confirmed that there was little difference in taste between samples when looking at PC1 as a standard. Therefore, although there are different components that affect taste according to the marketing age, it is judged that the taste difference between 28–34 day will not be felt overall.

### 3.7. Sensory Evaluation

Table 5 lists the results of the sensory evaluation of cooked cured chicken breast with various marketing ages. As indicated, no significant differences were observed in terms of the color, juiciness, taste, and overall acceptability, excluding the flavor and texture. In terms of the flavor, the cured chicken breasts with marketing ages of 30 and 32 day scored significantly higher than those with marketing ages of 28 and 34 day (*p* < 0.05). While the tenderness tended to decrease with increasing marketing age, no significant difference was observed between the ages of 28–32 day.

In this study, the lightness, redness, and yellowness values of the cooked cured chicken breast showed significant differences according to age, but no significant differences were observed in the visual evaluation of color according to age. As mentioned above, for the purpose of this study, sodium nitrite was added during the preparation of the curing solution. Products exhibiting the typical cured meat color resulting from the addition of sodium nitrite tend to be preferred by consumers due to their familiar color and uniform appearance [45]. However, in this case, although differences in the measured color values were detected, no significant visual differences resulted from the addition of sodium nitrite.

As described above, the flavor of raw chicken meat is determined by components such as the free amino acids and fatty acids [46], which can be influenced by various factors, including the breed, sex, age, and nutritional content of the feed provided in the breeding environment, although age tends to impart the greatest effect [20]. In general, for the same breed and variety, the meat of an older organism tends to possess a stronger aroma and flavor (metallic, bloody) compared to younger organisms [37], and so the determination of an optimal age for processing is necessary. As a result of the PCA analysis for evaluating differences in flavor of the cooked cured chicken breast in samples of different ages, no significant difference was observed between the ages of 30 and 32 day, although significant differences were observed between the ages of 28 and 34 day. Since the highest cured chicken breast flavor scores were obtained for the 30 and 32 day samples, marketing ages of 30 and 32 day were considered suitable for processing in terms of the flavor.

However, in terms of the texture, it was found that the tenderness decreased with increasing marketing age. This was attributed to the fact that, with increasing maturity, the structures of the extracellular fibers, such as collagen, elastin, and reticulin, become more dense, thereby resulting in an increased shear force, as mentioned above [20]. Such a reduction in tenderness therefore resulted in a low score for the texture component. Although the meat was tenderized through massaging with the curing solution during the tumbling process, the shear force increased, and the tenderness of the cured chicken breast decreased with age. Therefore, in terms of texture, a marketing age of 28–32 day was considered optimal for processing.

## 4. Conclusions

In this study, the quality properties of cured chicken breast manufactured using broiler chicken breast of various marketing ages (i.e., 28, 30, 32, and 34 day) were evaluated. It was found that increasing the broiler chicken marketing age resulted in the water holding capacity (WHC), protein content, pH, and shear force tending to increase. However, the 34 day sample exhibited different properties in terms of its color and aromatic profiles (as determined by principal component analysis). Furthermore, the flavor traits in the sensory evaluation of cooked cured chicken breast prepared from the 30 and 32 day samples were superior to those of the 28 and 34 day samples. It was therefore concluded that the current broiler marketing age of 32 day for cured chicken breast is suitable for the consumer and the Korean market.

## Figures and Tables

**Table 1:** Proximate composition of cooked cured chicken breast with various marketing ages.

Traits (%)	Marketing Ages (day)			
	28	30	32	34
Water	70.62 ± 0.10 ^a^	68.79 ± 0.77 ^a,b^	68.85 ± 0.09 ^a,b^	68.13 ± 1.26 ^b^
Protein	21.09 ± 2.53 ^b^	24.45 ± 0.17 ^a,b^	24.42 ± 0.64 ^a,b^	24.91 ± 0.34 ^a^
Fat	0.95 ± 0.12	1.00 ± 0.08	1.01 ± 0.07	1.11 ± 0.14
Ash	2.50 ± 0.04	2.50 ± 0.06	2.57 ± 0.14	2.48 ± 0.08

Data are shown as means ± SE. ^a–b^ Means on the same row with different letters are significantly different (*p* < 0.05).

**Table 2:** pH and water-holding capacity (WHC) of uncooked and cooked cured chicken breast with various marketing ages.

Traits		Marketing Ages (d)			
		28	30	32	34
pH	Uncooked	5.85 ± 0.01 ^c^	5.86 ± 0.01 ^c^	5.93 ± 0.01 ^b^	5.95 ± 0.01 ^a^
	Cooked	6.14 ± 0.01 ^d^	6.18 ± 0.01 ^c^	6.20 ± 0.01 ^b^	6.26 ± 0.01 ^a^
WHC (%)		90.17 ± 0.13 ^b^	94.98 ± 0.45 ^a^	94.02 ± 1.21 ^a^	96.09 ± 1.48 ^a^

Data are shown as means ± SE. ^a–d^ Means on the same row with different letters are significantly different (*p* < 0.05).

**Table 3:** Color of cooked cured chicken breast with various marketing ages.

Traits	Marketing Ages (day)			
	28	30	32	34
CIE L* (lightness)	70.68 ± 0.55 ^a^	69.90 ± 0.81 ^a^	67.22 ± 0.64 ^b^	63.70 ± 0.68 ^c^
CIE a* (redness)	3.83 ± 0.12 ^b^	3.82 ± 0.20 ^b^	3.77 ± 0.15 ^b^	4.65 ± 0.26 ^a^
CIE b* (yellowness)	18.25 ± 0.44 ^c^	19.19 ± 0.14 ^b^	19.27 ± 0.90 ^b^	20.40 ± 0.75 ^a^
Hue angle (H°)	74.14 ± 0.58 ^a^	78.74 ± 0.53 ^a^	78.90 ± 0.91 ^a^	77.16 ± 0.61 ^b^
Chroma (C*)	18.65 ± 0.42 ^c^	19.57 ± 0.17 ^b^	19.64 ± 0.86 ^b^	20.92 ± 0.76 ^a^

Data are shown as means ± SE. ^a–c^ Means on the same row with different letters are significantly different (*p* < 0.05).

**Table 4:** Taste score of cooked cured chicken breast with various marketing ages.

Items	Marketing Ages (day)			
	28	30	32	34
AHS_sourness ^(1)^	3.76	8.41	6.61	5.23
PKS	6.28	5.99	5.89	5.83
CTS_saltiness	5.38	5.87	5.99	6.76
NMS_umami	5.39	5.91	5.91	6.79
CPS	5.81	5.77	5.88	6.54
ANS	8.04	3.99	5.51	6.46
SCS	8.12	3.24	5.53	7.10

Data are shown as means. ^(1)^ Indicated the 7-sensor array of the electronic tongue.

**Table 5:** Sensory evaluation of cooked cured chicken breast with various marketing ages.

Traits	Marketing Ages (day)			
	28	30	32	34
Color	8.86 ± 0.42	9.04 ± 0.56	8.88 ± 0.39	8.96 ± 0.58
Flavor	8.36 ± 0.49 ^b^	9.30 ± 0.50 ^a^	9.24 ± 0.56 ^a^	8.22 ± 0.75 ^b^
Texture	9.20 ± 0.65 ^a^	8.92 ± 0.79 ^a,b^	8.90 ± 0.54 ^a,b^	8.56 ± 0.63 ^b^
Juiciness	8.82 ± 0.96	8.76 ± 0.58	8.70 ± 0.72	8.74 ± 0.61
Taste	8.80 ± 0.94	8.73 ± 0.60	8.68 ± 0.73	8.78 ± 0.64
Overall acceptability	8.66 ± 0.84	8.62 ± 0.68	8.70 ± 1.00	8.64 ± 0.91

Data are shown as means ± SE. ^a–b^ Means on the same row with different letters are significantly different (*p* < 0.05).
